# Specific immune-inflammatory profiles and neurocognitive deficits predict illness trajectories in people with type 2 diabetes mellitus or psychiatric disorders

**DOI:** 10.1016/j.bbih.2025.100962

**Published:** 2025-02-13

**Authors:** Joan Vicent Sánchez-Ortí, Patricia Correa-Ghisays, Vicent Balanzá-Martínez, Gabriel Selva-Vera, Víctor M. Victor, Constanza San Martin Valenzuela, Pau Soldevila-Matías, Fabián Robledo-Yagüe, María Flores-Rodero, Jon Sánchez-Valle, Jaume Forés-Martos, Diego Macías Saint-Gerons, Inmaculada Fuentes-Durá, Rafael Tabarés-Seisdedos

**Affiliations:** a INCLIVA - Health Research Institute, Valencia, Spain; b TMAP - Evaluation Unit in Personal Autonomy, Dependency and Serious Mental Disorders, University of Valencia, Valencia, Spain; c Center for Biomedical Research in Mental Health Network (CIBERSAM), Health Institute, Carlos III, Madrid, Spain; d Faculty of Psychology, University of Valencia, Valencia, Spain; e Teaching Unit of Psychiatry and Psychological Medicine, Department of Medicine, University of Valencia, Valencia, Spain; f VALSME (VALencia Salut Mental i Estigma) Research Group, University of Valencia, Valencia, Spain; g Department of Physiology, Faculty of Medicine and Dentistry, University of Valencia, Valencia, Spain; h Service of Endocrinology and Nutrition, University Hospital Dr. Peset, Valencia, Spain; i Foundation for the Promotion of Health and Biomedical Research in the Valencian Region (FISABIO), Valencia, Spain; j Department of Physiotherapy, University of Valencia, Valencia, Spain; k Department of Psychology, Faculty of Health Sciences, European University of Valencia, Valencia, Spain; l Computational Biology Group, Life Sciences Department, Barcelona Supercomputing Center, Barcelona, Spain; m Faculty of Nursery, University of Valladolid, Valladolid, Spain

**Keywords:** Illness trajectories, Neurocognition, Immune-inflammation, Type 2 diabetes mellitus, Schizophrenia, Bipolar disorder, Major depressive disorder

## Abstract

**Introduction:**

Psychiatric disorders and type 2 diabetes mellitus (T2DM) are chronic conditions that are often comorbid with each other. Neurocognitive and functional impairments are associated with numerous clinical changes during the course of illness. Immune-inflammatory dysfunction is emerging as a critical factor in the progression of these disorders. This study aimed to identify neurocognitive deficits and immune-inflammatory biomarkers that are suitable for signaling different illness trajectories from transdiagnostic and longitudinal perspectives.

**Methods:**

Clinical status, neurocognitive and functional performance, and peripheral blood biomarkers of immune-inflammation were assessed twice a year in 165 individuals, including 30 with schizophrenia (SZ), 42 with bipolar disorder (BD), 35 with major depressive disorder (MDD), 30 with T2DM, and 28 healthy controls (HCs). Participants with chronic illness (n = 137) were stratified into quartiles, taking their years of illness duration at baseline as a reference into categories of short illness duration (SD; n = 37), middle illness duration (MD; n = 36), long illness duration (LD; n = 32), and very long illness duration (VLD; n = 32). The illness duration was used to measure the illness trajectory, and the exposure of interest was clinical progression, calculated as the difference between clinical severity at baseline (T1) and after 1 year (T2).

**Results:**

Neurocognitive impairment was more significant in the VLD group than in the other groups, with small–moderate effect sizes (F = 2.9 to 9.3; p < 0.05−0.0001; η^2^p = 0.06−0.24). Moreover, the HC group showed significantly higher functional outcomes than the other groups (F = 5.8 to 6.0; p < 0.0001; η^2^p = 0.13−0.16). On the contrary, the HC group showed lower levels of immune-inflammatory markers (white blood cell count, absolute neutrophils, absolute monocytes, absolute basophiles, neutrophils/lymphocyte ratio, and platelets/lymphocyte ratio [PLR]) (F = 2.9 to 6.7; p < 0.05−0.0001; η^2^p = 0.07−0.18). In all groups, significant prospective associations were observed between cognitive function (short-term memory and processing speed), global functional scores, immune-inflammatory biomarkers (monocyte/lymphocyte ratio [MLR] and PLR), and clinical status (p < 0.05). Furthermore, a similar combination of neurocognitive deficits and immune-inflammatory alterations compounded the transdiagnostic model that best discriminated the different illness trajectories (χ^2^ = 67.4 to 78.7; p < 0.05−0.01).

**Conclusions:**

Neurocognitive dysfunction and systemic inflammation are associated with prolonged illness trajectories in individuals with psychiatric disorders and T2DM. An immune-inflammatory profile and neurocognitive and functional performance may be valuable to differentiate individuals with different illness trajectories. These findings have potential translational utility for early transdiagnostic interventions targeting these groups.

## Highlights

• Individuals with very long illness trajectory are characterized by increase of systemic inflammation and overall impairment in neurocognitive performance.
• Short-term memory, processing speed, global functional score, monocyte/lymphocyte ratio, platelets/lymphocyte ratio compounded an useful transdiagnostic model for predicting clinical progression across chronic illness groups, regardless of illness duration.
• Systemic inflammation is a key common factor in identifying individuals with stable disease trajectories from those with worsening/improving.
• Neurocognitive and functional performance is relevant in differentiating individuals with opposing disease trajectories, while its role in differentiating clinical stability from worsening or improvement is less significant.

## 1 Introduction

Psychiatric disorders and metabolic diseases manifest as chronic illnesses that often overlap. Type 2 diabetes mellitus (T2DM) is a common comorbidity among individuals with depressive disorder (MDD), bipolar disorder (BD), and schizophrenia (SZ) (Lindekilde et al., 2022). The co-occurrence of T2DM and psychiatric disorders is associated with worse clinical outcomes and more adverse illness trajectories (Kremers et al., 2022). In this context, clinical progression is considered to contribute to the significant multimorbidity reported in psychiatric disorders and the increased vulnerability to neurocognitive and functional impairment over time (Morris et al., 2019; Pacheco et al., 2022). Neuroprogression is the pathological reorganization of the central nervous system (CNS) that occurs when clinical, cognitive, and functional impairments are related to the illness course (Malhi et al., 2020). Neuroprogression encompasses the biological processes underlying the clinical progression of psychiatric disorders (Kupka et al., 2021).

In recent years, immune-inflammatory pathways have been shown to play a significant role in clinical progression. A recent review suggested that immune-inflammatory dysfunction could determine the recurrence and severity of mood episodes, treatment responses, and recovery in individuals with MDD (Debnath et al., 2021). Elevated inflammation can also negatively affect the clinical trajectory of BD, leading to an accelerated illness progression (Grewal et al., 2022). Similarly, a longer illness duration in individuals with SZ has been associated with higher counts of white blood cells (WBC) and other inflammatory markers (e.g., interleukins and C-reactive protein), especially during psychotic episodes, suggesting that immune-inflammatory processes have a relevant impact on the illness trajectory (Özdin and Böke, 2019). The neutrophil/lymphocyte ratio (NLR), monocyte/lymphocyte ratio (MLR), and platelet/lymphocyte ratio (PLR) are often used as indicators of inflammation in psychiatric disorders (Bhikram and Sandor, 2022; Jackson and Miller, 2020; Mazza et al., 2020). Likewise, a recent study highlighted the existence of an underlying predisposition of individuals with first psychotic episodes to present an increased mean NLR (Bioque et al., 2022).

All three inflammatory ratios (NLR, MLR, and PLR) have been used to predict the course of illness in many non-psychiatric disorders (He et al., 2022). Furthermore, insulin resistance is a source of systemic inflammation and immunological dysfunction in MDD, suggesting a bidirectional relationship in which metabolic comorbidities can affect clinical progression (Leonard and Wegener, 2020). It should be noted that inflammatory pathways related to insulin resistance have been proposed as critical factors for worse illness trajectories in individuals with BD and SZ (Bryll et al., 2020; Calkin et al., 2021). Alterations in blood cell counts have also been described in newly diagnosed naïve patients with non-affective psychosis compared to healthy controls (HCs) (Garcia-Rizo et al., 2019).

Increasing evidence shows that neurocognitive and functional impairments are common in highly comorbid disorders such as T2DM and psychiatric disorders, and are sometimes used as proxies for clinical progression (McCleery and Nuechterlein, 2019; Tomic et al., 2022). Longitudinal studies have shown that neurocognitive deficits are related to a worse illness course and suggest that they could be significant predictors of clinical status in individuals with psychotic and mood disorders (Herold et al., 2021; Valerio et al., 2020). Similarly, previous studies have correlated poor functional performance with adverse clinical trajectories (Hall et al., 2019; López-Villarreal et al., 2020). Furthermore, the presence of T2DM can exacerbate neurocognitive impairments associated with comorbid diseases (Li et al., 2020). Increasing evidence suggests that progressive impairment of neurocognitive and functional performance could be related to disrupted immune-inflammatory pathways (Borovcanin et al., 2020; Cuperfain et al., 2020).

To the best of our knowledge, no studies have evaluated the predictive ability of immune-inflammatory biomarkers and neurocognitive and functional performance in detecting worse illness trajectories in people with psychiatric disorders or T2DM. Ideally, such studies should be based on both transdiagnostic and longitudinal perspectives. This study aimed to compare immune-inflammatory biomarkers and neurocognitive and functional performance in individuals with psychiatric disorders or T2DM, stratified by illness duration, to explore whether baseline immune-inflammatory biomarkers and neurocognitive and functional performance are significant predictors of illness trajectory, and to examine the usefulness of immune-inflammatory biomarkers and neurocognitive and functional performance to detect a worse illness trajectory at 1-year follow-up.

## 2 Materials and methods

### 2.1 Study design and ethical considerations

This study is part of a project that aims to identify and validate peripheral biomarkers of neurocognitive deficits in patients with MDD, BD, SZ, and T2DM. This prospective comparative cohort study was conducted between April 2015 and January 2018 to investigate the association and evolution of specific peripheral blood biomarkers and neurocognitive impairment in a unique longitudinal cohort of individuals with somatic and psychiatric disorders. Demographic and clinical data, neurocognitive and functional data, and information on peripheral blood biomarkers were collected at baseline (T1) and after 1 year (T2). Individuals with psychiatric disorders were recruited from mental health units (MHUs) in several towns in the province of Valencia, Spain (Gandía, Foios, Catarroja, Paterna, and Sagunto), the psychiatry outpatient clinic, and the endocrinology department of the University Hospital Dr. Peset and Miguel Servet MHU in Valencia City. HCs were residents of the same area as those with SMI. Participants were demographically matched. Written informed consent was obtained from all participants after the study procedure was fully explained. The Ethics Committees and Institutional Review Boards of each participating center approved the study protocol and the ethical principles of the Declaration of Helsinki were followed. Only variables related to the objectives of the present study were included in the analyses.

### 2.2 Participants

SZ, BD, and MDD were diagnosed according to the criteria of the Diagnostic and Statistical Manual of Mental Disorders 5 (DSM-5) (American Psychiatric Association, 2014). T2DM was diagnosed according to the American Diabetes Association Standards of Care (ADA, 2015). Participants with MDD and BD were required to meet the remission criteria (Tohen et al., 2009) for an acute affective episode. Individuals with SZ also met the remission criteria for psychotic episode (Andreasen et al., 2005). Individuals with T2DM were required to be free from severe diabetic neuropathy and kidney disease (serum creatinine <1.5 mg/dL). Moreover, we checked the fasting glucose levels in the individuals with psychiatric disorder and HCs to reduce possible biases attributable to overlapping of the glycemic pathway between these individuals and those with T2DM. This check was carried out prior to the cognitive and functional assessments at T1 and T2. For the recruitment of HCs, the absence of physical illness, pharmacological treatment, and a family history of psychiatric disorders in first-degree relatives was required. Ability to understand study procedures and willingness to provide written consent were required for participation. General exclusion criteria for all groups included current hospitalization; documented cognitive impairment not secondary to psychiatric disorders such as intellectual disability or significant neurocognitive disorders, i.e., dementia; disability or inability that prevented understanding of the protocol; current substance use disorders (except nicotine); pregnancy; intake of steroids, corticosteroids, antioxidants, antibiotics, and immunologic therapies; fever with temperature over 38 °C; and history of vaccination within 4 weeks of evaluation. The same inclusion and exclusion criteria were used for patients with T1 and T2 disease.

### 2.3 Clinical and neuropsychological assessment

The same experienced psychologists and psychiatrists of the research group conducted all the assessments. Sociodemographic data, including sex, age, years of education, and motor laterality (defined as manual, ocular, and crural dominance), were collected.

Clinical evaluations were conducted using the following instruments: (i) 17-item Hamilton Rating Scale for Depression (HDRS) (Ramos-Brieva and Cordero-Villafáfila, 1986), (ii) Young Mania Rating Scale (Colom et al., 2002), (iii) Positive and Negative Syndrome Scale (PANSS) (Peralta and Cuesta, 1994), (iv) Kaplan−Feinstein Scale (KFS) (Rosas-Carrasco et al., 2011), (v) Charlson Comorbidity Index (CCI) (Rius et al., 2004), and (vi) Clinical Global Impression (CGI) scale (Haro et al., 2003; Vieta et al., 2002). The total number of prescribed psychopharmacological and other medications was also recorded.

There are no consensus-based clinical thresholds for the categorization of the illness course; therefore, participants were stratified by quartiles, taking their years of illness duration at baseline as a reference (de la Fuente-Tomas et al., 2019; Zghebi et al., 2019). The distribution by quartile was: >1 year to ≤7 years [quartile-1 group: short illness duration (SD)], >7 years to ≤15 years [quartile-2 group: middle illness duration (MD)], >15 years to ≤25 years [quartile-3 group: long illness duration (LD)], >25 years [quartile-4 group: very long illness duration (VLD)]. The cut-off point for chronic disease was ≥1 year. This cut-off point was established because chronicity has high individual variability related to disease symptoms and functional performance, which in turn strongly impacts perceived well-being (Malhi et al., 2020). The lack of an objective criterion for this question led us to opt for a statistical measure (minimum value of the variable “number of years of duration”) that would circumvent the subjectivity of this construct as much as possible.

The exposure of interest was clinical progression in each participant with chronic illness, calculated as the difference between the CGI at T1 and T2 as a measure of clinical progression. It was categorized into “stable disease trajectory (SDT) = 0”, “worsening disease trajectory (WDT) ≤ -1”, and “improving disease trajectory (IDT)≥1”. The CGI measured at both time points was continuously and categorically examined (Busner et al., 2009; Dunlop et al., 2017).

Cognitive performance was evaluated using a comprehensive battery of neuropsychological tests and subtests previously used by our group (Aliño-Dies et al., 2020; Correa-Ghisays et al., 2017, 2019, 2022; Garés-Caballer et al., 2022; Luperdi et al., 2021; Salazar-Fraile et al., 2009; San Martin-Valenzuela et al., 2020; Sánchez-Ortí et al., 2022, 2023, 2024; Selva-Vera et al., 2010; Tatay-Manteiga et al., 2018; Tabarés-Seisdedos et al., 2008). The battery included the premorbid intelligence quotient, which was calculated using the Wechsler Adult Intelligence Scale III edition (WAIS-III) Vocabulary subtest, considered a classical measure of intelligence level before the onset of a mental disorder (Krull et al., 1995). Cognitive reserve (CR) was estimated based on the results of the WAIS-III Vocabulary subtest and number of years of formal education (Lyman, 1971). Seven cognitive domains were assessed: (i) learning and verbal memory (L&VM): Complutense Verbal Learning Test (TAVEC) for total immediate recall, short-term free recall, and long-term free recall variables (Benedet and Alejandre, 2014); (ii) cognitive flexibility (CF): Stroop Color and Word test (SCWT) color/word subtest (Golden, 2001) and Wisconsin Card Sorting Test categories for completed and perseverative errors (Grant and Berg, 2001); (iii) verbal fluency (VF): FAS and animal-naming test for phonemic and semantic fluency, respectively (Reitan and Wolfson, 1985); (iv) working memory (WM): Trail-Making Test (TMT) Part B (Reitan and Wolfson, 1985) and WAIS-III digit span backward (Weschler and Memory, 1999); (v) short-term memory (StM): TAVEC immediate recall of the first learning trial, immediate recall of the interference list (Benedet and Alejandre, 2014), and WAIS-III digit span forward (Weschler and Memory, 1999); (vi) visual memory (VM): Rey−Osterrieth Complex Figure Test (ROCFT) 2 min after the copy (fRey2) and 20 min after the copy (fRey20) (Rey, 1999); and (vii) processing speed (PS): finger-tapping test left unimanual, right unimanual, left bimanual, and right bimanual and average of the four scores (Reitan and Wolfson, 1985; Tabarés-Seisdedos et al., 2003), WAIS-III digit symbol coding subtest (Weschler and Memory, 1999), SCWT color and word subtests (Golden, 2001), and TMT Part A (Reitan and Wolfson, 1985). The global cognitive score (GCS) was calculated by averaging the scores of seven cognitive domains.

Functional performance was evaluated using the Functional Assessment Short Test (FAST) (Rosa et al., 2007), Short Form-36 Health Survey questionnaire (SF-36) (Alonso et al., 1995), and World Health Organization Quality of Life Brief Scale (WHO-QoL-Bref) (Bobes et al., 2004). The global functional score (GFS) was calculated by averaging the total scores on three scales.

The study used the validated Spanish version of all clinical, cognitive, and functional assessment instruments.

### 2.4 Determination of immune-inflammatory biomarkers in blood

Venous blood extraction was performed, and serum and plasma samples were stored in a freezer at −80 °C. Citrated blood samples were incubated with dextran (3%) for 45 min to isolate human polymorphonuclear leukocytes (PMNs). The supernatant was dropped over Ficoll-Hypaque (GE Healthcare, Barcelona, Spain) and centrifuged for 25 min at room temperature at 650×*g*. Lysis buffer was added to the remaining erythrocytes in the pellet, which was incubated at room temperature for 5 min and then centrifuged at 240×*g* for 5 min. The PMNs were washed twice and resuspended at 37 °C in Hanks’ balanced salt solution (HBSS; Sigma Aldrich, MO). Scepter 2.0 cell counters (Millipore, MA, USA) were used to count the cells. The fraction of peripheral mononuclear blood cells was obtained using the BD Vacutainer® CPT™ Mononuclear Cell Preparation Tube - Sodium Citrate (BD Biosciences, NJ, USA). Each tube was centrifuged at 1800 rpm for 25 min to separate plasma from red and white cells, after which the fraction corresponding to white cells was centrifuged at 2500 rpm for 10 min at 4 °C; the supernatant was discarded and the pellets were washed with PBS.

The following blood count parameters were taken into account: WBC, neutrophils (WBC-N), absolute neutrophils (WBC-AN), lymphocytes (WBC-L), absolute lymphocytes (WBC-AL), monocytes (WBC-M), absolute monocytes (WBC-AM), eosinophils (WBC-E), absolute eosinophils (WBC-AE), basophiles (WBC-B), absolute basophiles (WBC-AB), platelets (PLT), mean corpuscular volume (MDV), and inflammatory ratios (NLR, MLR, and PLR).

### 2.5 Statistical analyses

Data were analyzed using the Statistical Package for Social Sciences (version 26.0; IBM Corp., 2019). The sample size was calculated using Ene 2.0 software, which estimated that 29 individuals were sufficient to ensure representativeness (Pedromingo-Marino, 2005).

In the pre-analysis phase, we standardized and normalized the scores obtained in the questionnaires that made up the functional performance assessment (FAST, SF-36, and WQB), taking as a reference the maximum scores of each questionnaire and the values obtained by the control group. Given that the three questionnaires measure slightly different constructs, we performed a cluster analysis to verify that the constructs could be grouped within a global construct, which we called GFS. We also inverted the scores of the questionnaires in those cases where it was necessary to perform the calculation correctly (e.g., FAST). Similarly, we proceeded with the scales that make up the GCS construct. Likewise, potential confounding variables (e.g., psychiatric symptoms and comorbidities) interference with illness trajectories, GCS, GFS, and biomarkers was previously explored.

Descriptive analyses were performed using one-way analysis of variance (ANOVA) for continuous variables and the chi-square test for categorical variables. Differences between the groups in CGI, neurocognitive and functional performance, and immune-inflammatory biomarkers at T1 and T2, and their evolution over time were assessed using mixed multivariate analysis of covariance (MANCOVA), with age and years of education as covariates. Normality was assumed for all continuous variables because the sample was statistically verified using the Kolmogorov−Smirnov test. This ensured that MANOVA/MANCOVA parametric tests could be used at both times. A post-hoc analysis with Bonferroni-corrected pairwise *t*-test and Mann−Whitney *U* test was performed to examine group differences. The effect sizes were calculated with partial eta-squared (η^2^p), and the following values were used as a reference: small = 0.02, moderate = 0.15, and large = 0.35. The direct scores obtained for neurocognitive and functional performance were transformed into z-scores. The mean and standard deviation of the HCs in T1 were used as reference values to calculate the z-scores.

To test the predictive ability of immune-inflammatory biomarkers and neurocognitive and functional performance at T1 to discriminate individuals with different illness trajectories over time, a binary logistic regression was performed using a predictive model that included only immune-inflammatory biomarkers and neurocognitive and functional performances that were statistically significant for each group according to clinical progression. Subsequently, a receiver operating characteristic (ROC) curve was generated for the variables identified in the binary logistic regression as explanatory factors of the different illness trajectories. The analysis showed that the area under the ROC curve for the identification of individuals with LD/VLD was 74.5% (95% CI = 0.66–0.83).

Similarly, a linear regression analysis was performed using a predictive model that included only statistically significant outcomes for each group to test the predictive ability of immune-inflammatory biomarkers and neurocognitive and functional performance at baseline to explain the variance in clinical progression at T2. Other relevant variables (age, comorbidities, mood, and psychotic symptoms) related to clinical progression was not included because they were not the focus of this study and immune-inflammatory biomarkers and neurocognitive and functional outcomes were considered optimal predictors. Statistical significance was set at p < 0.05. The procedure for generating predictive models is as follows: First, a predictive analysis was performed on each biomarker separately and predictive models were generated that included statistically significant variables (p < 0.05). Finally, the optimal predictive combination was obtained.

## 3 Results

### 3.1 Sample description

At T1, the sample consisted of 165 individuals: 30 with SZ, 42 with BD, 35 with MDD, 30 with T2DM, and 28 HCs. Participants with chronic illness (n = 137) were stratified into quartiles, taking as a reference their years of illness duration at baseline, comprising 37 in the SD group (T2DM = 9, MDD = 17, BD = 4, and SZ = 7), 36 in the MD group (T2DM = 11, MDD = 8, BD = 7, and SZ = 10), 32 in the LD group (T2DM = 7, MDD = 3, BD = 13, and SZ = 9), and 32 in the VLD group (T2DM = 3, MDD = 7, BD = 18, and SZ = 4).

In total, 40 participants were lost to follow-up at T2 (T2DM = 25, MDD = 25, BD = 29, and SZ = 27, HCs = 9) (retention rate, 75.7%). At T2, the sample consisted of 21 individuals in the SD group (T2DM = 6, MDD = 11, BD = 1, and SZ = 3), 26 in the MD group (T2DM = 9, MDD = 5, BD = 3, and SZ = 9), 30 in the LD group (T2DM = 7, MDD = 2, BD = 9, and SZ = 12), 29 in the VLD group (T2DM = 3, MDD = 7, BD = 16, and SZ = 3), and 19 HCs.

A summary of the baseline sociodemographic and clinical characteristics of the participants in each group (SD, MD, LD, VLD, and HCs) is shown in Table 1. Females represented 48% of the participants. The mean age of the total sample was 46.2 (SD, 12.3) years. The HC group was characterized as significantly younger and had more years of education. Psychiatric symptoms, multimorbidity, and the average/mean number of non-psychopharmacological medications were similar between the chronic illness groups. The SD group used a significantly lower number of psychopharmacological medications than the VLD group.

**Table 1:** Sociodemographic and clinical characteristics of the sample at T1.

Variables^a^	HC	SD	MD	LD	**VLD**	***Statistical analyses***	
	(*n* = 28)	*(n* = 37)	(*n* = 36)	(*n* = 32)	(*n* = 32)	F(*p*)^g^^,^^i^	Post hoc test^h^^,^^j^
*Sociodemographic*							
Sex^b^	18(64%)	14(38%)	12(33%)	19(59%)	17(53%)	NS	
Age	36.6(14.5)	42.6(14.0)	47.3(10.8)	50.8(9.9)	56.2(5.8)	12.8∗∗∗∗	HC < MD,LD,VLD SD,MD < VLD
Years of education	16.1(3.3)	12.5(4.6)	11.5(4.5)	11.2(4.5)	11.2(4.6)	6.5∗∗∗∗	VLD.LD,MD,SD < HC
Laterality^c^	23(82%)	32(86%)	34(94%)	31(96%)	30(93%)	NS	
*Clinical*							
SMI^d^	–	28(75%)	25(69%)	25(78%)	29(91%)	NS	
HDRS^e^	–	7.5(6.7)	6.9(6.1)	5.7(4.1)	9.5(7.7)	NS	
YMRS^e^	–	2.7(3.5)	2.1(4.2)	2.5(3.3)	3.1(4.5)	NS	
PANSS-P^e^	–	8.0(2.9)	8.3(3.4)	8.1(2.7)	8.6(3.9)	NS	
PANSS-N^e^	–	9.3(5.7)	12.7(10.5)	11.0(7.3)	10.9(5.8)	NS	
PANSS-G^e^	–	20.7(8.0)	25.3(14.5)	22.1(9.1)	23.0(8.8)	NS	
KFS	–	1.1(1.6)	1.3(1.7)	1.7(2.1)	2.1(1.9)	NS	
CCI	–	1.1(1.5)	1.2(1.6)	1.1(1.6)	1.4(1.4)	NS	
Psychiatric medicines^f^	–	1.8(1.7)	2.5(2.4)	2.7(1.8)	3.4(2.1)	3.5∗	SD < VLD
General medicines^f^	–	3.6(3.2)	4.4(2.4)	5.0(3.0)	4.5(2.3)	NS	

a Expressed as mean(standard deviation) except when indicated.

b female n(%).

c right-handers n(%).

d severe mental illness n(%).

e lower scores represent a better outcome.

f number.

g ANOVA.

h Chi-squared test.

i Bonferroni test.

j Mann-Whitney *U* test. Abbreviations: HC = healthy control, SD = short-illness duration, MD = middle-illness duration, LD = long-illness duration, VLD = longest-illness duration, SMI = severe mental illness, HDRS = Hamilton rating scale for depression, YMRS = Young mania rating scale, PANSS = positive and negative syndrome scale, P = positive, N = negative, G = general, KFS = Kaplan-Feinstein scale, CCI = Charlson comorbidity index, NS = not significant. (NS = p > 0.05; ∗p ≤ 0.05; ∗∗p ≤ 0.01; ∗∗∗p ≤ 0.001; ∗∗∗∗p ≤ 0.0001).

### 3.2 Between-group comparison of clinical course and neurocognitive and functional performance

The clinical course, neurocognitive function, and functional performance of all groups at T1 and T2 are presented in Table 2. The severity of the clinical course was significantly higher for the VLD and LD groups compared to the SD group at T1 (p < 0.001; η^2^p = 0.11) and for the MD and SD groups at T2 (p < 0.0001; η^2^p = 0.19). Regarding cognitive domains, compared to the HC group, the VLD group showed significantly lower performance in CF, WM, StM, VM, and PS at T1 (p < 0.05−0.0001; η^2^p = 0.06−0.18) and L&VM at T2 (p < 0.05−0.0001; η^2^p = 0.09−0.24). Similarly, the LD group significantly underperformed HCs in StM and PS at both times (p < 0.01−0.0001; η^2^p = 0.09−0.24), and the MD group showed significantly lower performance than HCs in StM at both times (p < 0.01−0.0001; η^2^p = 0.09−0.21). Furthermore, individuals with VLD exhibited significantly lower performances in CF, WM, and PS than those with MD and SD at T1 (p < 0.0001; η^2^p = 0.14−0.18) and those with SD at T2 (p < 0.05−0.0001; η^2^p = 0.09−0.24). GCS was significantly lower in the VLD group than in the other groups at T1 (p < 0.0001; η^2^p = 0.17) and at T2, except for the LD group. Moreover, individuals with LD showed significantly lower neurocognitive performance than SD and HCs at T2 (p < 0.0001; η^2^p = 0.20). In contrast, GFS was significantly higher in HCs than in all clinical groups at both times (p < 0.0001; η^2^p = 0.13−0.16). Small-to-moderate effect sizes were observed for clinical course and neurocognitive and functional performance in the between-group comparisons at both assessments. No significant within-group differences were observed in neurocognitive or functional performance over time.

**Table 2:** Clinical course, neurocognitive and functional outcomes. Between-group comparison at T1 and T2.

	HC		**SD**		MD		LD		VLD		*Statistical analyses*					
Variables^a^	**T1** (*n* = 28)	**T2** (*n* = 19)	**T1***(n* = 37)	**T2** (*n* = 21)	**T1** (*n* = 36)	**T2** (*n* = 26)	**T1** (*n* = 32)	**T2** (*n* = 30)	T1 (*n* = 32)	T2 (*n* = 29)	T1 F(*p*)^c^	Post hoc test^d^	η^2^p^e^	T2 F(*p*)^c^	Post hoc test^d^	η^2^p^e^
*Clinical course*																
CI^b^	–	–	2.9(1.5)	2.8(1.7)	3.4(1.5)	3.3(1.7)	3.5(1.1)	3.8(1.1)	3.5(1.0)	4.0(1.2)	5.7∗∗∗	SD < LD,VLD	0.11	7.9∗∗∗∗	SD < LD,VLD MD < VLD	0.19
*Cognitive domains*																
L&VM	0.0(1.0)	0.8(1.1)	−0.7(1.4)	−0.1(1.2)	−1.3(1.4)	−0.8(1.5)	−1.2(1.4)	−1.1(1.5)	−1.6(1.2)	−1.1(1.2)	NS			4.7∗∗∗	VLD,LD < SD,HC	0.14
CF	0.0(1.0)	0.3(1.0)	−1.0(1.2)	−0.4(1.2)	−1.4(1.8)	−1.3(1.6)	−1.6(1.3)	−1.8(1.4)	−2.8(1.3)	−2.5(1.6)	7.2∗∗∗∗	VLD < LD,MD,SD,HC	0.15	6.2∗∗∗∗	VLD,LD < SD,HC	0.17
VF	0.0(1.0)	0.2(1.1)	−0.5(1.2)	−0.4(1.2)	−0.6(1.2)	−0.5(1.1)	−0.8(1.3)	−0.6(1.1)	−0.9(1.0)	−0.8(0.8)	NS			NS		
WM	0.0(1.0)	0.4(0.8)	−1.6(2.3)	−0.8(1.1)	−1.9(2.4)	−1.9(2.6)	−2.6(3.0)	−2.6(3.2)	−5.0(4.1)	−3.5(3.0)	6.6∗∗∗∗	VLD < LD,MD,SD,HC	0.14	3.0∗	VLD < SD,HC	0.09
StM	0.0(1.0)	0.8(1.2)	−0.9(1.1)	−0.8(1.1)	−1.4(1.0)	−1.0(1.2)	−1.3(0.8)	−1.6(0.9)	−1.6(1.1)	−1.3(1.1)	3.9∗∗	VLD,LD,MD < HC	0.09	8.0∗∗∗∗	VLD,LD,MD,SD < HC	0.21
VM	0.0(1.0)	0.6(0.8)	−1.2(1.5)	−0.3(1.5)	−1.3(1.6)	−1.0(1.6)	−1.7(1.6)	−1.3(1.4)	−2.3(1.5)	−2.1(1.4)	2.9∗	VLD < HC	0.06	6.8∗∗∗∗	VLD,LD < HC VLD < SD	0.19
PS	0.0(1.0)	0.1(1.0)	−1.1(1.3)	−1.0(0.8)	−1.6(1.4)	−1.5(1.3)	−2.3(1.5)	−2.3(1.5)	−3.0(1.3)	−2.8(0.9)	8.8∗∗∗∗	VLD < MD,SD,HC LD < HC	0.18	9.3∗∗∗∗	VLD < MD,SD,HC LD < SD,HC	0.24
CR	0.0(1.0)	0.1(1.1)	−0.4(1.5)	−0.5(1.6)	−0.6(1.5)	−0.7(1.4)	−0.8(1.4)	−0.8(1.5)	−0.7(1.6)	−0.7(1.6)	NS			NS		
*Neurocognitive and functional performance*																
GCS	0.0(1.0)	0.5(1.0)	−1.8(2.0)	−1.0(1.6)	−2.3(2.2)	−2.0(2.3)	−3.0(2.4)	−2.9(2.5)	−4.5(2.5)	−3.7(1.9)	7.9∗∗∗∗	VLD < LD,MD,SD,HC	0.17	7.6∗∗∗∗	VLD < MD,SD,HC	
LD < SD,HC	0.20															
GFS	0.0(1.0)	0.2(0.5)	−2.2(2.2)	−2.1(2.6)	−2.4(2.2)	−2.1(2.5)	−2.8(1.8)	−2.4(1.8)	−2.9(2.3)	−3.3(2.2)	6.0∗∗∗∗	VLD,LD,MD,SD < HC	0.13	5.8∗∗∗∗	VLD,LD,MD,SD < HC	0.16

a Z-scores expressed as mean(standard deviation).

b lower scores represent a better outcome.

c MANCOVA.

d Bonferroni test.

e Partial Eta-Squared (η^2^p). Abbreviations: HC = healthy control, SD = short-illness duration, MD = middle-illness duration, LD = long-illness duration, VLD = longest-illness duration, T1 = time 1, T2 = time 2, CI = clinical impression, L&VM = learning and verbal memory, CF = cognitive flexibility, VF = verbal fluency, WM = working memory, StM = short-term memory, VM = visual memory, PS = processing speed, CR = cognitive reserve, GCS = global cognitive score, GFS = global functional score, NS = not significant. (NS = p > 0.05; ∗p ≤ 0.05; ∗∗p ≤ 0.01; ∗∗∗p ≤ 0.001; ∗∗∗∗p ≤ 0.0001). Effect size (η^2^p: small ≈ 0.02; moderate ≈ 0.15; large ≈ 0.35).

### 3.3 Between-group comparison of immune-inflammatory biomarkers

The peripheral biomarkers at T1 and T2 in the five groups are shown in Table 3. Overall, HCs showed significantly lower levels of immune-inflammatory biomarkers at both time points. WBC and WBC-AN were significantly higher in the chronic illness groups than in the HC group at both times (p < 0.01; η^2^p = 0.09−0.17). Similarly, the HC group exhibited significantly lower scores in WBC-AB, NLR, and PLR at T1 (p < 0.01−0.0001; η^2^p = 0.09−0.12) and T2, except for the NLR biomarker (p < 0.05; η^2^p = 0.09). In contrast, WBC-AL was significantly higher in SD compared to the VLD, LD, and HC groups at both times (p < 0.001; η^2^p = 0.10−0.15). In all cases, the effect size ranged from small to moderate. Within-group differences across time were not significant for any biomarker in any group.

**Table 3:** Immune-inflammatory profile. Between-group comparison at Time 1 and Time 2.

	HC		**SD**		MD		LD		VLD		*Statistical analyses*					
Variables^a^	**T1** (*n* = 28)	**T2** (*n* = 19)	**T1***(n* = 37)	**T2** (*n* = 21)	**T1** (*n* = 36)	**T2** (*n* = 26)	**T1** (*n* = 32)	T2 (*n* = 30)	T1 (*n* = 32)	T2 (*n* = 29)	T1 F(*p*)^b^	Post hoc test^c^	η^2^p^d^	T2 F(*p*)^b^	Post hoc test^c^	η^2^p^d^
WBC	6.5(1.6)	6.2(1.3)	8.1(2.3)	8.8(2.7)	7.9(1.9)	8.2(1.9)	7.5(2.3)	7.7(2.2)	7.5(1.2)	7.5(1.6)	4.2∗∗	HC < VLD,LD,MD,SD	0.10	6.2∗∗∗∗	HC < VLD,LD,MD,SD	0.17
WBC-N	54.0(7.7)	53.9(8.8)	56.2(7.9)	53.9(8.3)	58.1(7.6)	56.1(11.5)	59.9(7.7)	60.0(9.5)	59.8(9.0)	56.7(9.8)	NS			NS		
WBC-AN	3.5(1.1)	3.3(1.1)	4.6(1.8)	4.8(2.2)	4.7(1.5)	4.7(1.5)	4.6(1.9)	4.7(1.8)	4.4(1.1)	4.5(1.2)	4.1∗∗	HC < VLD,LD,MD,SD	0.09	4.2∗∗	HC < VLD,LD,MD,SD	0.12
WBC-L	35.5(6.7)	35.2(8.6)	33.9(7.2)	34.6(7.7)	30.9(7.1)	32.9(11.0)	28.7(6.5)	29.3(8.0)	28.5(9.2)	31.5(8.4)	3.6∗∗	VLD,LD < HC	0.08	NS		
WBC-AL	2.2(0.6)	2.1(0.5)	2.7(0.7)	2.9(0.7)	2.4(0.6)	2.5(0.9)	2.1(0.5)	2.1(0.5)	2.0(0.6)	2.0(0.8)	4.7∗∗∗	VLD,LD,HC < SD	0.10	5.2∗∗∗	VLD,LD,HC < SD	0.15
WBC-M	9.2(6.5)	7.6(1.3)	7.3(1.4)	8.2(1.9)	7.4(2.0)	8.0(1.3)	7.3(1.4)	7.5(1.9)	7.1(1.7)	7.4(1.9)	NS			NS		
WBC-AM	0.5(0.1)	0.4(0.1)	0.6(0.2)	0.6(0.1)	0.5(0.1)	0.6(0.1)	0.5(0.1)	0.5(0.1)	0.5(0.1)	0.5(0.1)	3.1∗∗	HC < SD	0.07	6.7∗∗∗∗	HC < SD,MD	0.18
WBC-E	2.8(1.9)	2.6(1.3)	2.2(0.9)	2.6(1.1)	2.5(1.3)	2.2(1.4)	2.5(1.2)	2.6(1.2)	3.0(1.6)	3.0(1.9)	NS			NS		
WBC-AE	0.1(0.1)	0.1(0.1)	0.1(0.1)	0.2(0.1)	0.2(0.1)	0.1(0.1)	0.2(0.1)	0.1(0.1)	0.2(0.1)	0.2(0.1)	NS			NS		
WBC-B	0.5(0.2)	0.5(0.2)	0.4(0.1)	0.6(0.2)	0.5(0.1)	0.5(0.2)	0.5(0.2)	0.6(0.3)	0.5(0.2)	0.6(0.2)	NS			NS		
WBC-AB	0.01(0.02)	0.01(0.03)	0.02(0.03)	0.04(0.04)	0.04(0.03)	0.04(0.03)	0.04(0.03)	0.05(0.05)	0.04(0.03)	0.05(0.03)	3.8∗∗	HC < MD,LD,VLD	0.09	3.1∗	HC < LD,VLD	0.09
PLT	215.3(51.3)	212.5(52.9)	235.7(53.1)	253.8(52.4)	241.5(62.8)	239.6(50.1)	246.6(73.1)	247.5(85.2)	262.3(66.0)	249.6(44.2)	NS			NS		
MDV	9.8(1.1)	9.9(1.2)	10.0(1.0)	9.9(0.8)	10.2(1.3)	10.2(1.1)	10.5(1.3)	10.3(1.0)	10.6(1.4)	10.4(1.2)	NS			NS		
NLR	1.6(0.5)	1.6(0.6)	1.7(0.6)	1.6(0.6)	2.0(0.9)	2.0(1.2)	2.2(1.0)	2.3(1.1)	2.4(1.2)	2.0(0.9)	3.6∗∗	HC < LD,VLD SD < VLD	0.08	NS		
MLR	0.2(0.1)	0.2(0.07)	0.2(0.05)	0.2(0.08)	0.2(0.09)	0.2(0.1)	0.2(0.05)	0.2(0.07)	0.2(0.1)	0.2(0.08)	NS			NS		
PLR	6.2(1.7)	6.1(1.4)	7.3(2.6)	7.6(2.4)	8.3(3.8)	8.1(3.5)	9.2(4.3)	9.2(4.1)	9.9(3.2)	8.9(2.7)	5.5∗∗∗∗	HC < LD,VLD SD < VLD	0.12	2.9∗	HC < LD,VLD	0.09

a Expressed as mean(standard deviation).

b MANCOVA.

c Bonferroni test.

d Partial Eta-Squared (η^2^p). Abbreviations: HC = healthy control, SD = short-illness duration, MD = middle-illness duration, LD = long-illness duration, VLD = longest-illness duration, T1 = time 1, T2 = time 2, WBC = white blood cell, N = neutrophils, AN = absolute neutrophils, L = lymphocytes, AL = absolute lymphocytes, M = monocytes, AM = absolute monocytes, E = eosinophils, AE = absolute eosinophils, B = basophiles, AB = absolute basophiles, PLT = platelets, MDV = mean corpuscular volume, NLR = neutrophil/lymphocyte ratio, MLR = monocyte/lymphocyte ratio, PLR = platelet/lymphocyte ratio, NS = not significant. (NS = p > 0.05; ∗p ≤ 0.05; ∗∗p ≤ 0.01; ∗∗∗p ≤ 0.001; ∗∗∗∗p ≤ 0.0001). Effect size (η^2^p: small ≈ 0.02; moderate ≈ 0.15; large ≈ 0.35).

### 3.4 Predictive ability of immune-inflammatory biomarkers and neurocognitive and functional performance at T1 of the clinical progression at T2

The relative contributions of immune-inflammatory biomarkers and neurocognitive and functional performance at T1 to clinical progression at T2 are shown in Table 4. For the entire sample, the combination of performance in the two cognitive domains (StM and PS), GFS, and immune-inflammatory biomarkers (MLR and PLR) significantly predicted clinical progression at T2, explaining 55.4% of the variance. Furthermore, the model prediction was significant when tested separately in each group and explained between 15.9% and 64.5% of the clinical progression variance at T2 in the chronic illness groups.

**Table 4:** Predictive immune-inflammatory biomarkers and neurocognitive, functional performance at T1 of global clinical progression at T2.

Dependent variables at T2	Predictors at T1 associated	*β*	95% CI	*t*	Percent of variance explained (adjusted *R*^*2*^)
**CP**	**StM**	−0.19	−0.47 to −0.04	2.4∗	55.4
	**PS**	−0.24	−0.43 to −0.07	2.8∗∗	
	**GFS**	−0.45	−0.44 to −0.23	6.4∗∗∗∗	
	**MLR**	−0.12	−5.79 to 0.19	1.9∗	
	**PLR**	0.14	0.008 to 0.13	2.2∗	

Abbreviations: T1 = time 1, T2 = time 2, CP = clinical progression, StM = short-term memory, PS = processing speed, GFS = global functional score, MLR = monocyte/lymphocyte ratio, PLR = platelets/lymphocyte ratio. Significant level: ∗p ≤ 0.05; ∗∗p ≤ 0.01; ∗∗∗p ≤ 0.001; ∗∗∗∗p ≤ 0.0001.

### 3.5 Discriminatory ability of immune-inflammatory biomarkers and neurocognitive functional performance at T1 to differentiate individuals with different illness trajectories at 1-year follow-up

**Fig. 1:**
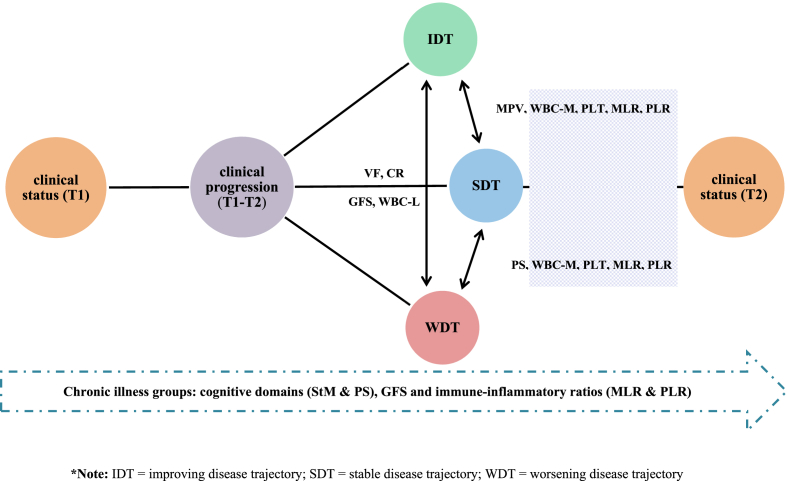

The ability of immune-inflammatory biomarkers and neurocognitive and functional performance at T1 to differentiate different illness trajectories at the 1-year follow-up is presented in Table 5. The combination of cognitive performance in PS and immune-inflammatory biomarkers (WBC-M, PLT, MLR, and PLR) resulted in a model that best discriminated individuals with SDT from those with WDT at 1-year follow-up, with a correct differentiation rate of 67.4%. There was a positive correlation between clinical progression and MLR (p < 0.05) and a negative correlation between clinical progression and PS (p < 0.05), WBC-M (p < 0.05), and PLR (p < 0.05). Similarly, the combination of only immune-inflammatory biomarkers (WBC-M, PLT, MPV, MLR, and PLR) best differentiated individuals with SDT from those with IDT at 1-year follow-up, with a correct classification rate of 78.7%. There was a positive relationship between clinical progression and MLR (p < 0.05), and a negative correlation between clinical progression and WBC-M (p < 0.05) and PLR (p < 0.05). In contrast, the combination of cognitive domains (VF and CR), GFS, and WBC-L were the models that best differentiated individuals with WDT from those with IDT at the 1-year follow-up, with a correct classification rate of 71.7%. There was a positive relationship between CR (p < 0.05) and a negative correlation with VF (p < 0.05), GFS (p < 0.05), and WBC-L (p < 0.05) (Fig. 1). The analysis showed that the area under the ROC curve for the identification of individuals with WDT was 83.9% (95% CI = 0.77–0.91) compared to SDT and 83.9% (95% CI = 0.77–0.91).

**Table 5:** Immune-inflammatory biomarkers and neurocognitive, functional performance at T1 with ability to discriminate individuals with different illness trajectory at 1-year follow-up.

Dependent variables	Predictors at T1 associated	*β*	Wald	Percent of variance explained	Global percentage correctly predicted
**CP (SDT vs. WDT)**	**PS**	−0.37	3.7∗	14%–20%	67.4
	**WBC-M**	−1.02	3.5∗		
	**PLT**	0.03	4.8∗		
	**MLR**	36.82	5.0∗		
	**PLR**	−0.95	4.7∗		
**CP (SDT vs. IDT)**	**WBC-M**	−1.45	4.3∗	13%–20%	78.7
	**PLT**	0.04	5.5∗∗		
	**MDV**	0.62	3.4∗		
	**MLR**	43.16	4.5∗		
	**PLR**	−1.35	5.3∗		
**CP (WDT vs. IDT)**	**VF**	−1.02	4.2∗	22%–30%	71.7
	**CR**	0.59	3.5∗		
	**GFS**	−0.47	5.5∗∗		
	**WBC-L**	−0.10	3.3∗		

Abbreviations: T1 = time 1, T2 = time 2, CP = clinical progression, SDT = stable disease trajectory, WDT = worsening disease trajectory, IDT = improving disease trajectory, VF = verbal fluency, PS = processing speed, CR = cognitive reserve, GFS = global functional score, WBC = white blood cell, L = lymphocytes, M = monocytes, PLT = platelets, MDV = mean corpuscular volume, MLR = monocyte/lymphocyte ratio, PLR = platelet/lymphocyte ratio. Significant level: ∗p ≤ 0.05; ∗∗p ≤ 0.01; ∗∗∗p ≤ 0.001; ∗∗∗∗p ≤ 0.0001.

## 4 Discussion

To our knowledge, this is the first study that aimed to identify immune-inflammatory biomarkers as well as neurocognitive and functional performance, which are useful to detect different disease trajectories across individuals with psychiatric disorders and T2DM, using a longitudinal design and transdiagnostic perspective.

Our findings show an overall impairment in neurocognitive performance along with an increase in systemic inflammation in individuals with a very long illness trajectory. As expected, functional performance decreased in all clinical groups. Regarding progression, immune-inflammatory activity and neurocognitive and functional performance were critical to predict clinical course across chronic illness groups, regardless of illness duration. Systemic inflammation was a key common factor in identifying and differentiating individuals with stable illness trajectories from those with worsening or improvement at 1-year follow-up. Moreover, neurocognitive and functional performance is relevant in distinguishing individuals with opposite illness trajectories, whereas its role in differentiating clinical stability from worsening or improvement is less apparent. Overall, our findings are consistent with the neuroprogressive hypothesis of psychiatric disorders (Kupka et al., 2021).

The present results are consistent with previous evidence suggesting that neurocognitive impairment is strongly related to the illness trajectory in psychiatric disorders (Jonas et al., 2022; Melloni et al., 2019). For example, attention, memory, and VF are more affected in individuals with first-episode psychosis, while executive function is more impaired in individuals with chronic SZ (Kelly et al., 2019). These cognitive deficits are potential predictors of the severity of clinical symptoms and can determine illness trajectory (Habtewold et al., 2020). Similarly, neurocognitive impairment significantly influences clinical progression in individuals with BD (Sánchez-Morla et al., 2019) and MDD (Yang et al., 2021). In fact, it has been postulated to be a critical predictor of differences in the clinical trajectories of individuals with mood disorders (Burdick and Millett, 2021; Liu et al., 2023). A recent review also suggested that a longer diabetes trajectory accelerates neurocognitive impairment and increases the risk of developing dementia (van Duinkerken and Ryan, 2020), so neurocognitive impairment may help identify individuals with T2DM characterized by worse clinical progression (Ji et al., 2023). Our results are also consistent with previous findings suggesting that functional impairment has a negative impact on the course of illness in people with psychiatric disorders and T2DM (de Winter et al., 2022; TODAY Study Group, 2022). Emerging evidence suggests that functional performance is a critical factor in differentiating illness trajectories and can contribute to optimizing preventive measures in these individuals (Bjornstad et al., 2023; Montelongo et al., 2023). In summary, accumulative evidence appears to support the proposition that overlapping neurocognitive and functional impairments significantly influence disease progression and lead to different illness trajectories (McWhirter et al., 2020; Rønne et al., 2020).

Concomitantly, immune-inflammatory activity is a key characteristic of disease progression. The results of our study are consistent with previous findings, suggesting that common immune-inflammatory pathways are strongly related to clinical severity across psychiatric disorders (Byrne et al., 2022; Kwon and Cheon, 2021). Emerging evidence suggests that proinflammatory activity promotes the development of negative symptoms in individuals with psychosis (Goldsmith et al., 2019) and is associated with depressive symptoms in individuals with mood disorders (Huang et al., 2023), thus correlating with poorer clinical outcomes. Inflammation also appears to underlie neurodegenerative processes related to illness progression in individuals with metabolic diseases, such as T2DM (Kacířová et al., 2020). Our results also show that immune-inflammatory mechanisms can play a key role in predicting poorer clinical outcomes over time and converge with those of recent studies (Cuperfain et al., 2020; Debnath et al., 2021; Halstead et al., 2023). For instance, monocyte, pro-inflammatory interleukin (IL-6), and C-reactive protein (CRP) levels were negatively associated with the clinical stage in a sample of individuals with SZ (Feng et al., 2020) and increased risk of progression over time (Adamowicz et al., 2024). Similarly, decreased levels of monocytes and proinflammatory factors (IL-6, CRP, and tumor necrosis factor-alpha [TNF-a]) are associated with poorer clinical outcomes in BD (Karthikeyan et al., 2022) and MDD (Moriarity et al., 2020). Recent evidence has shown that differences in immune profiles can help identify individuals with unipolar and bipolar depression (Brunoni et al., 2020) and those with worse illness trajectories (Poletti et al., 2021). Moreover, an impaired immune-inflammatory profile can increase vulnerability to T2DM (Blériot et al., 2023) and has been postulated to be a significant predictor of poorer clinical outcomes over time (Sluiman et al., 2022). Therefore, the immune-inflammatory response may be a promising therapeutic target in individuals with a worse prognosis (Al-Fadhel et al., 2020; Stassen et al., 2021).

These findings are clinically relevant. It is recommended to implement early intervention, effective strategies targeting neurocognitive and functional impairments, and prevention of immune-inflammatory alterations. Therefore, people at risk of developing worse illness trajectories can be identified and treated more intensively than those who are not expected to deteriorate over time. Longitudinal studies with longer follow-up periods and larger sample sizes are required to replicate these findings. Similarly, it would be desirable to include other biomarkers (e.g., metabolism, oxidative stress, and neurotrophins) to validate the potential effect of exacerbations on the clinical course of psychiatric disorders and T2DM.

This study has several limitations. First, the relatively small sample size reduces the generalizability of the results to populations with clinical characteristics similar to those studied. Second, high sample attrition was observed after a year of follow-up, and, in turn, the follow-up period was short. This may have led to a potential bias in retaining individuals who completed the assessments and, therefore, were in a better clinical condition. Third, the immune-inflammatory mechanisms among participants could fluctuate depending on their clinical status and pharmacological treatment (Tatay-Manteiga et al., 2018). Furthermore, the potential role of lifestyle behaviors (Ringin et al., 2022; Van Rheenen and O'Neil, 2022) was not considered in the present analysis. Fourth, the third objective was to examine the complex relationship between illness trajectory and clinical progression. Furthermore, a longer duration of the disease does not always imply a worsening clinical course (Rybakowski, 2021). Finally, our choice of the cut-off point for considering the disease as chronic was arbitrary, as the minimum value of the variable “years of disease duration” was chosen as a criterion. Despite these limitations, this study included a novel transdiagnostic approach and a comprehensive assessment of immune-inflammatory biomarkers and neurocognitive and functional performance in individuals stratified by illness duration across populations with T2DM and psychiatric disorders. Moreover, the longitudinal design allowed the establishment of potential causal relationships between the immune-inflammatory profile, neurocognitive performance, functional outcomes, and clinical progression. In addition to these limitations, this study included a novel transdiagnostic approach and a comprehensive assessment of cognitive and functional outcomes in individuals stratified by illness duration in populations with T2DM and psychiatric disorders. Moreover, the longitudinal design allowed for the establishment of potential causal relationships between adiposity-related inflammation and neurocognitive and functional outcomes. Finally, the multicenter design of this study may have increased the external validity of our results.

## 5 Conclusion

In conclusion, our findings emphasize the critical role of shared immune-inflammatory pathways in different disease trajectories. Similar neurocognitive and functional impairments are emerging as essential players in illness progression. The co-occurrence of these mechanisms may lead to a worse clinical course. These data provide new clinical approaches to address the progression of psychiatric disorders and T2DM. Managing the immune-inflammatory profile and identifying different neurocognitive and functional trajectories may help maintain stable clinical trajectories in these disorders.

## CRediT authorship contribution statement

**Joan Vicent Sánchez-Ortí:** Writing – review & editing, Writing – original draft, Methodology, Investigation, Formal analysis, Data curation. **Patricia Correa-Ghisays:** Writing – review & editing, Writing – original draft, Methodology, Investigation, Formal analysis, Data curation. **Vicent Balanzá-Martínez:** Writing – review & editing, Supervision, Resources, Project administration. **Gabriel Selva-Vera:** Writing – review & editing, Resources, Methodology, Investigation. **Víctor M. Victor:** Writing – review & editing, Resources, Methodology, Investigation. **Constanza San Martin Valenzuela:** Writing – review & editing, Resources, Methodology, Investigation. **Pau Soldevila-Matías:** Writing – review & editing, Supervision, Methodology, Investigation. **Fabián Robledo-Yagüe:** Writing – review & editing, Resources, Methodology, Investigation. **María Flores-Rodero:** Writing – review & editing, Resources, Methodology, Investigation. **Jon Sánchez-Valle:** Writing – review & editing, Resources, Methodology, Investigation. **Jaume Forés-Martos:** Writing – review & editing, Resources, Methodology, Investigation. **Diego Macías Saint-Gerons:** Writing – review & editing, Resources, Methodology, Investigation. **Inmaculada Fuentes-Durá:** Writing – review & editing, Resources, Methodology, Investigation. **Rafael Tabarés-Seisdedos:** Writing – review & editing, Supervision, Resources, Project administration.

## Funding

This work was supported by 10.13039/501100004587Carlos III Health Institute (ISCIII) 10.13039/100005622Health Research Fund (FIS) grants [grant ID: PI22/00424, PI19/0838, PI17/00719, and PI14/00894]. European Regional Development Fund (10.13039/501100008530ERDF “A way to build Europe”) and the Ministry of Education of the Valencian Regional Government [grant ID: PROMETEO/2019/027, CIPROM/2022/58, and CIPROM/2022/32], 10.13039/501100004837Spanish Ministry of Science and Innovation [grant ID: CB06/04/0071 and PID2021-129099OB-I00], and 10.13039/501100004828Grand Challenges Canada [grant ID: 0456-03-40].

## Declaration of competing interest

Dr. V. Balanzá-Martínez has received honoraria from Angelini over the last 3 years unrelated to the present work.

## Data Availability

Data will be made available on request.
